# Role of Serum Ferritin as a Predictor of Dengue Severity: A Prospective Observational Study From India

**DOI:** 10.7759/cureus.63503

**Published:** 2024-06-30

**Authors:** Pawan K Goyal, Kanika Hissaria, Chanchal Shekhawat

**Affiliations:** 1 Internal Medicine, Fortis Hospital, New Delhi, IND; 2 Family Medicine, Jaipur Golden Hospital, New Delhi, IND; 3 Internal Medicine, Jaipur Golden Hospital, New Delhi, IND

**Keywords:** hospital stay, platelet count, serum ferritin, severe fever, dengue

## Abstract

Background: Early detection of dengue fever progression to severe form using simple measurable tests is crucial for initiating appropriate supportive therapy. This study aimed to assess whether serum ferritin is an indicator of severity in dengue patients.

Material and methods: This hospital-based prospective observational study was done on 70 patients admitted in wards and intensive care unit (ICU) of Aditya Birla Memorial Hospital, Pune, India, and Jaipur Golden Hospital, Delhi, India, satisfying inclusion criteria during the study period. Dengue cases were classified as those who had non-structural protein 1 (NS1) positivity on days 2-8 and/or positive immunoglobulin M (IgM) on days 6-10, while those with other confirmed diagnoses were considered as other febrile illnesses. The outcome of this study was to see an association between serum ferritin levels and severity of illness, condition at the time of discharge/death, and the length of hospitalization.

Results: Mean serum ferritin (ng/mL), platelet count (cells/mm^3^), and hospital stay (in days) were 1469.43±297.53, 17289.29±8254.47, and 6.01±3.90, respectively. Subjects with severe dengue had significantly higher mean serum ferritin levels and median hospital stays compared to those with non-severe dengue (p<0.05). A moderate (Pearson correlation coefficient ρ=-0.35, p=0.0027) negative correlation was found between serum ferritin level and platelet count whereas a moderate (ρ=0.50, p=0.0000) positive correlation was found between serum ferritin level and hospital stay.

Conclusion: Serum ferritin levels can be used as a tool to help differentiate between severe and non-severe dengue.

## Introduction

*Aedes aegypti* and *Aedes albopictus* mosquitoes transmit a viral infection called dengue, a serious health concern in many regions [1]. Nearly 50-100 million new infections worldwide are reported by the World Health Organization (WHO), with 50,000 cases of severe dengue requiring hospitalization and causing mortality in nearly 2.5% of individuals [2]. The number of cases has increased nearly eight-fold over the last 20 years from 505,430 cases in 2000 to about 5.2 million in 2019. In 2012, the WHO recognized it as "the most significant viral disease spread by mosquitos" [3]. Dengue fever has a case fatality rate of 3-5% in some rural regions of India [4]. Despite simple and inexpensive management, the fever becomes life-threatening if not diagnosed in its initial stage. It is widely established that dengue causes substantial cytokine activation. Different dengue virus (DV) serotypes I-IV induce symptoms ranging from asymptomatic infection to severe dengue. Dengue fever is suspected when a febrile illness lasting two to seven days comprises two or more symptoms such as headache, retro-orbital pain, muscle pain, joint pain, rash, and hemorrhagic presentation [5]. For the first five days of fever, NS1 antigen reactivity by enzyme-linked immunosorbent assay (ELISA) technique is used to diagnose dengue fever. Within two to three days of sickness, NS1 has a diagnostic sensitivity of more than 90%. However, the sensitivity steadily declines, and it decreases drastically after the fifth day [6]. Dengue virus-specific IgM may be detected and used to diagnose DV with high sensitivity and specificity. Those who have never been infected with dengue virus have a slow IgM response that increases by 50% after three to five days, 80% after more than five days, and 100% after 10 days [7,8]. Severe dengue is classified as dengue hemorrhagic fever and dengue shock syndrome by WHO [9]. Since the severe form has no specific treatment, early detection and proper medical care could lower the fatality rate to below 1%. As a result, several biomarkers for immunological and endothelial cell activity have been established to predict the severity [10]. Dengue infection causes an increase in acute-phase reactants such as alpha1 antitrypsin, ceruloplasmin, and ferritin. The reticuloendothelial system produces ferritin, an iron storage protein complex of isoferritins. The clinical reaction of depriving bacteria of serum iron is reflected in elevated levels of serum ferritin, an acute-phase reactant [11]. Hyperferritinemia in dengue-infected people is linked to severe immunological activation and coagulation problems. Earlier reports have revealed a correlation between serum ferritin and the outcome (lower serum ferritin levels correlating with better outcome). The serum ferritin levels have been linked to severe dengue fever in children [12]. This study included 177 children with serum ferritin levels assessed throughout the clinical course. This study concluded that serum ferritin levels greater than or equal to 1,200 ng/mL may predict dengue hemorrhagic fever.

A study conducted on Aruba Island during a dengue outbreak reported that ferritin can be used as an effective clinical marker for differentiating dengue from other febrile diseases [13]. Serum ferritin levels exceeding 1,500 ng/mL were associated with severe illness, thus it is vital to constantly monitor patients who have hyperferritinemia, which is associated with significant immunological activation and coagulation issues [13].

It is critical to forecast the risk of severe dengue fever as soon as possible using basic quantifiable tests, so that necessary, intense supportive care may be started. Our study aimed to determine if serum ferritin measured during the early course of the disease can predict dengue severity, which could aid in triage and management appropriately. The study further aimed to see a correlation between serum ferritin levels and the duration of hospital stay.

## Materials and methods

This hospital-based prospective observational study was conducted at Aditya Birla Memorial Hospital, Pune, India, and Jaipur Golden Hospital, Delhi, India, to determine the association between serum ferritin levels and the severity of dengue illness, condition at the time of discharge, and the length of hospitalization. The sample size was determined using the effect size from a previous study and the following formula: n=z^2 ^pq/d^2^, where p=0.769 (an estimation of the sensitivity of elevated serum ferritin levels to predict severe dengue), q=0.231 (compliment of "p"), z=1.96 (score at 95% confidence interval), and d^2^=0.10 (margin of error) [14]. Thus, according to this formula, the minimum sample size required was 68, which we rounded off to 70. Data were collected from 70 patients admitted to wards and ICUs between December 2020 and April 2021. Although this was the period of the COVID-19 pandemic, strict protocols as prescribed by the applicable guidelines were followed to avoid any impact on the conduct of the study. Further, the study did not include any COVID-19-positive patients. The study included male and female patients who met the WHO 2012 definition of dengue fever. Febrile patients with serology-positive dengue from outside labs were also included in the study. Patients were excluded if they refused to give consent for participation in the study or were pregnant. Participants who met the inclusion and exclusion criteria and gave informed consent to participate in the study were enrolled. The ethical approval for the study was obtained from the Institutional Ethics Committee and Scientific Research Committee of Jaipur Golden Hospital, New Delhi, with approval number JGH/DNB/MD/2020/2408 dated December 12, 2020.

Patients with NS1 positivity on days two to eight and/or positive IgM on days six to 10 were defined as dengue cases; those with other confirmed diagnoses were labeled as other febrile illnesses. Severe dengue was marked by severe thrombocytopenia, significant bleeding, plasma leakage, fluid buildup, respiratory distress, and multi-organ failure [5]. Other cases were marked as non-severe dengue. Inpatients admitted with undiagnosed causes of fever, serum levels of ferritin, total leukocyte count (TLC), platelet count, and hematocrit were measured. Dengue serology was followed up for positivity. Dengue immunoglobulin G (IgG) and IgM were estimated in the hospital laboratories by micro-ELISA kit and NS1 positivity was also estimated by ELISA kit. Serum ferritin levels were estimated on the day of admission by the immuno-luminescence method by Cobas-E411 Instrument (Penzberg, Germany: Roche Diagnostics GmbH). Hemoglobin, TLC, differential count, platelet, and hematocrit were estimated by photometric evaluation with auto-analyzer LH 750, 780 (Brea, CA: Beckman Coulter, Inc.). The outcome of this study was to see an association between serum ferritin levels and severity of illness, condition at the time of discharge/death, and the length of hospitalization.

The data was recorded in a predesigned proforma and managed on an Excel spreadsheet. The statistical analysis of the quantitative variables was summarized as arithmetic means with standard deviation (SD). Frequencies and percentages were used to summarize categorical and nominal data. The Student's t-test was used to compare the mean between the two groups for serum ferritin. Other two quantitative variables, i.e., platelet count and length of hospital stay, were non-normally distributed, therefore summarized as median (min-max); and, the Wilcoxon rank-sum test was used to compare distribution between the two groups. The Spearman's rank correlation was used to determine the strength of the relationship between serum ferritin, platelet count, and length of hospital stay. All statistical tests used were two-sided. Statistical analysis was performed using Stata 16.0 statistical software (College Station, TX: StataCorp LLC).

## Results

Of 70 subjects, 46 (65.71%) and 24 (34.29%) were male and female, respectively, with a majority belonging to the age groups of 21-40 years (70.00%). The mean age among the study subjects was 31.87±8.48 years. Severe dengue was reported in 17 (24.29%) subjects and non-severe dengue was reported in 53 (75.71%) subjects. The severe dengue manifestations observed were bleeding in 14 (82.35%) subjects, shock in 10 (58.82%) subjects, organ dysfunction in seven (41.18%) subjects, and respiratory distress in three (17.65%) subjects, respectively. The types of bleeding observed were petechiae, epistaxis, and gum bleeding, which required symptomatic management only without any need for platelet or blood transfusion. The mean values of serum ferritin (ng/mL), platelet count (cells/mm^3^), and hospital stay (in days) are enumerated in Table 1.

**Table 1:** Descriptive characteristics of the study population.

Variable	All (n=70)
Age, mean±SD, years	31.87±8.48
<20 years	11 (15.71)
21-40 years	49 (70.00)
>40 years	10 (14.29)
Sex, n (%)	
Female	24 (34.29)
Male	46 (65.71)
Dengue severity, n (%)	
Severe dengue	17 (24.29)
Non-severe dengue	53 (75.71)
Manifestations among severe dengue patients, n (%)	
Bleeding	14 (82.35)
Shock	10 (58.82)
Organ dysfunction	7 (41.18)
Respiratory distress	3 (17.65)
Serum ferritin, mean±SD (ng/mL)	1469.43±297.53
Platelet count, mean±SD (cells/mm^3^)	17289.29±8254.47
Hospital stay, mean±SD (days)	6.01±3.90

There were no reported deaths in the study and all patients were discharged from the hospital alive. The results revealed that mean serum ferritin level (p=0.0000) and median hospital stay (p=0.0000) were significantly higher among subjects suffering from severe dengue as compared to the subjects having non-severe dengue. Furthermore, the median platelet count was significantly higher (p=0.0000) in subjects suffering from non-severe dengue (Table 2).

**Table 2:** Comparison of serum ferritin levels, platelet count, and duration of hospital stay among severe and non-severe dengue cases. *P-values considered significant. Student’s t-test (for normally distributed continuous variables) and Wilcoxon rank-sum test (for non-normally distributed continuous variables) were used.

Parameters	Severe dengue		Non-severe dengue		p-Value
	Mean±SD	Median	Mean±SD	Median	
Serum ferritin (ng/mL)	1892.76±217.48	-	1333.64±159.78	-	0.0000*
Platelet count (cells/mm^3^)	-	8,516 (6,987-15,408)	-	15,462 (8,414-39,119)	0.0000*
Hospital stay (days)	-	12 (6-15)	-	4 (1-15)	0.0000*

A moderate negative correlation (Pearson correlation coefficient ρ=-0.35, p=0.0027) was found between serum ferritin level and platelet count, while a moderate positive correlation (ρ=0.50, p=0.0000) was found between serum ferritin level and hospital stay (Table 3).

**Table 3:** Correlation of serum ferritin levels with platelet count as well as duration of hospital stay. *P-values considered significant. Spearman's rank correlation coefficient test was used.

Parameters	Serum ferritin (ng/mL)	
	ρ-Value	p-Value
Platelet count (cells/mm^3^)	-0.35	0.0027*
Hospital stay (days)	0.50	0.0000*

## Discussion

With an estimated 3.9 billion people in 128 countries at risk of infection, dengue represents a significant public health concern [15]. Each year, around 400 million people worldwide are infected and 22,000 people die of severe dengue disease [16]. At present, there are no tests that can monitor or predict the severity and outcome of dengue. Therefore, we aimed to assess an association between serum ferritin level and severity of dengue illness to open new avenues for better targets as treatment modalities. The correlation between serum ferritin and the outcome was studied in a tertiary care hospital. In our study, the majority of the population were males which is in line with the previous studies done by Selvamuthukumaran and Kanitkar et al. [9,17]. The mean age of the study participants in our study was consistent with the findings of Nadeem et al. and Kanitkar et al. [17,18]. The bleeding, shock, organ dysfunction, and respiratory distress in severe dengue subjects were in line with the observations of a study by Diwakar and Madhu [19]. Of the 28 patients with severe dengue, they reported 24 patients with bleeding symptoms, 14 with shock, 13 with organ dysfunction, five with altered mental status, and five with respiratory distress.

Our study revealed significantly (p<0.05) higher mean serum ferritin levels and median hospital stays in patients with severe dengue. Previous studies have demonstrated a strong association between the development of severe dengue fever and elevated serum ferritin levels on the day of admission [10]. Serum ferritin levels in 104 dengue-positive patients were evaluated on the day of admission, and the patients were divided into the following two groups based on their serum ferritin levels: normal (up to 100 μg/dL) and high [20]. Both groups were monitored for the onset of severe dengue. Only two out of 31 individuals with normal serum ferritin levels developed severe dengue, whereas 35 out of 73 patients with severe dengue had elevated serum ferritin levels on the day of admission. The study's findings suggest that serum ferritin may be utilized as an early marker to predict the severity of the illness. Compared to non-severe cases, patients with severe dengue infection have increased expression of acute phase reactants. This helps recognize dengue-infected patients long before any clinical warning symptoms manifest. Ferritin is one such acute phase reactant that is produced by reticuloendothelial cells in reaction to inflammation and infection. Compared to individuals with other febrile disorders, people with dengue infection had significantly higher ferritin levels. The hyperferritinemia observed in these patients serves the following two purposes: in the early stages, elevated serum ferritin levels chelate the harmful free iron radicals at the site of inflammation, providing protection; while in severe cases, elevated ferritin may become pathogenic by inducing cytokine storm by activating immune cells [5]. In corroboration to our result, Petchiappan et al. [21] reported a direct association between hyperferritinemia and severe dengue, with a rise in serum ferritin levels starting on day four of illness. In a study conducted by Nadeem et al., patients with severe dengue fever exhibited significantly higher mean serum ferritin levels of 317.54±109.52 ng/mL, than patients with uncomplicated dengue fever (168.69±130.7 ng/mL) [18]. Similar observations were also made by Kanitkar et al. [17]. Furthermore, a study by Soundravally et al. found that high ferritin levels could possibly be able to predict the severity of dengue on the day of admission, with 76.9% sensitivity and 83.3% specificity [14]. In the current study, there was a statistically significant difference (p<0.05) in the median platelet count between participants with non-severe dengue and those with severe dengue. A moderate negative correlation was found between serum ferritin level and platelet count while a moderate positive correlation was found between serum ferritin level and hospital stay.

Limitation

There are several limitations to this study. First, the study relies on a single measurement of serum ferritin levels upon admission, which may not capture the dynamic changes in ferritin levels during the course of illness. Further, due to the small sample size, we were unable to establish the ferritin levels, which may be used to forecast the likelihood of developing severe dengue. Additionally, dengue serotypes and the type of infection (primary or secondary) were not taken into account. Also, the study did not adequately control for potential confounding variables such as underlying health conditions, nutritional status, or co-infections, which can affect serum ferritin levels. Further research with a larger sample size of severe cases would be required before a firm conclusion could be drawn.

## Conclusions

In conclusion, serum ferritin levels can be used as a tool to help differentiate between severe and non-severe dengue. Furthermore, platelet count has a negative correlation with serum ferritin levels, and duration of hospital stay has a positive correlation with serum ferritin levels.
